# Caregivers' feeding practices in Ethiopia: association with caregiver and child characteristics

**DOI:** 10.1017/jns.2021.14

**Published:** 2021-04-05

**Authors:** Nardos W. Gebru, Seifu H. Gebreyesus, Esete Habtemariam, Robel Yirgu, Dawit S. Abebe

**Affiliations:** 1 Department of Nutrition and Dietetics, School of Public Health, Addis Ababa University, 9086 Addis Ababa, Ethiopia; 2 Department of Nursing and Health Promotion, Oslo Metropolitan University, Postboks 4, St. Olavs, 0130 Oslo, Norway

**Keywords:** Children, Child BMI, Concern, Ethiopia, Feeding practices, Perception

## Abstract

Feeding is a source of interaction and communication. It affects children's physical and psychological/emotional development. The present study aims to examine the association between caregiver and child characteristics and caregivers' feeding practices among preschools in Addis Ababa. We conducted a cross-sectional study among 542 caregivers of children aged between 3 and 6 years old in selected preschools. We used the Child Feeding Questionnaire (CFQ) to measure caregivers' feeding practices. Multiple linear regression was used for analysis. Caregivers who had higher levels of perceived feeding responsibility (*β* 0⋅20, *P* < 0⋅001), who were more concerned about their child being overweight (*β* 0⋅11, *P* < 0⋅001) and who had more depressive symptoms (*β* 0⋅23, *P* 0⋅05) were associated with food restriction practice. Caregivers who were less concerned about their child being overweight (*β* −0⋅10, *P* < 0⋅001) and who had higher levels of perceived feeding responsibility (*β* 0⋅25, *P* < 0⋅001) were associated with pressure to eat practice. Caregivers who had higher education (*β* 0⋅29, *P* < 0⋅05), who had higher levels of perceived feeding responsibility (*β* 0⋅47, *P* < 0⋅001), who were more concerned about their child being overweight (*β* 0⋅15, *P* < 0⋅001) and who were less concerned about their child underweight (*β* −0⋅06, *P* < 0⋅05) were associated with monitoring feeding practice. In addition, as the children have gotten older (*β* 0⋅08, *P* < 0⋅05), there is increased use of monitoring feeding practice. This study is one of few studies that show the association between caregiver and child characteristics and feeding practices in developing countries such as Ethiopia. It is essential to include responsive feeding components in national nutritional programmes to improve preschool children's nutritional status in Ethiopia.

Feeding is a process that helps develop strong verbal and non-verbal interaction between caregivers/parents and children^(1)^. It affects children's physical and psychological/emotional development^(1)^. Parental/caregivers' feeding practices are food-specific strategies that parents/caregivers use to determine which foods the child should eat, where to eat and when to eat, and the emotional manner^(2)^. Parental/caregivers use different strategies in their feeding practices. One is pressure to eat, where parents/caregivers pressure their children to eat healthy food and maintain an adequate food intake^(3,4)^. Another feeding strategy is restriction, which involves attempting to control a child's diet by limiting his/her access to food, particularly unhealthy food. It is practiced directly to be detected by the child^(3,4)^. Another strategy is the monitoring feeding strategy, which involves parents/caregivers overseeing their child's eating^(2)^. It is a way of controlling a child's intake indirectly that cannot be detected by the child^(3)^.

It is vital to study caregivers' feeding practices in children's preschool years since the interaction between parents/caregivers and their children gradually develops and children become more autonomous compared to an earlier age^(5)^. Also, evidence indicates that children's eating behaviours are established in the preschool period and maintained later^(6)^.

Feeding difficulties in children are commonly reported by parents/caregivers. However, as many families experience non-clinical feeding problems, they do not seek clinical solutions, and matters like these are less likely to be identified and treated. Thus, common feeding difficulties can have an adverse effect on both the parent/caregiver and the child's physical and psychological health^(7)^. Caregivers' feeding practices also play an essential role in developing children's eating behaviours and subsequent weight status^(8–10)^. Controlling feeding practices (e.g. parental/caregivers' use of pressure to eat or restriction) have been linked to poorer appetite regulation, lower preference for fruit, and vegetable consumption, high intake of sugar-sweetened beverages, palatable snack foods and energy-dense food items^(11–13)^. Additionally, some studies have found pressure to eat to be associated with lower child weight status^(14,15)^, while other studies have found restriction to be related to higher child weight status^(15,16)^.

Most developing countries are currently facing a double burden of malnutrition and a surge in overweight and obesity^(17)^. Selecting adequate and appropriate foods is an essential factor in preventing malnutrition^(18)^. A recent study found that four out of ten children were not receiving an adequately diverse diet^(19)^. This finding was supported by national figures, indicating that children in Ethiopia have a monotonous diet, with more than 80 % of children not receiving adequate diet diversity^(20)^. A recent study conducted in Addis Ababa also found that food approach (i.e. enjoyment of food and food responsiveness) behaviours were positively associated with caregivers' practice of food restriction. In contrast, food avoidant behaviours (i.e. food fussiness and satiety responsiveness) were positively associated with caregivers' practice of pressure to eat^(21)^. These findings indicate the need to further investigate how children's eating behaviours and caregivers' controlling feeding practice contribute to poor nutritional status. Evidence suggests that feeding practices during the first years of childhood influence food intake, nutritional status and growth^(18)^. Unfortunately, these practices are often neglected, and very few studies have investigated feeding practices/styles adopted by caregivers and their effects^(18,22)^. In recent studies conducted in rural parts of Ethiopia, negative feeding behaviours were dominant and associated with very low food intake and stunted growth in children^(18,22)^. By contrast, another study conducted in southern Ethiopia (Derashe district) found that responsive feeding was the most common style practiced by the studied caregivers^(23)^.

Culture, socioeconomic circumstances and other related contexts play a pivotal role in shaping feeding practices, eating behaviours and perceptions of ‘healthy weight’^(24)^. Studies have shown that greater parental/caregiver concern about child underweight is positively associated with pressuring children to eat^(25)^, and those parents/caregivers who were highly concerned about their child becoming overweight were more likely to use restriction^(3)^. Cities like Addis Ababa are currently experiencing rapid urbanisation and socioeconomic and lifestyle changes that are continually modifying both access to food and feeding practices^(26)^. It has been found that urban mothers' decisions on what to feed their child are mainly based on child preferences, the effects of advertising and affordability or financial availability of food supply^(26)^. These continuously evolving food choices and lifestyle changes may affect how parents/caregivers feed their children.

Caregivers with depressive symptoms tend to display attitudes such as lack of interest or have difficulties concentrating and tend to be disengaged in caregiver–child interaction, including the use of less child-centred/controlling feeding practices^(27)^. Studies have shown that symptoms of maternal depression were associated with the higher reported use of controlling feeding practices^(28,29)^. Maternal depression is also related to mothers' use of food as a reward, practices that can disrupt children's self-regulation^(30)^. In Ethiopia, the prevalence of maternal depression is as high as 23⋅3 %^(31)^. A recent study showed that maternal depression was significantly associated with inappropriate complementary feeding and stunting^(32)^. However, there is a scarcity in the literature on associations between caregiver depressive symptoms and preschool children's nutritional status, eating behaviour or feeding practice.

Children's emotional and behavioural problems tend to become more pronounced during preschool age. This is due to the effect of these psychological problems on children's academic performance and peer relationships^(33)^. Children with psychological problems also make feeding interaction difficult since they tend to have impaired eating skills, such as hypersensitivity to taste and texture, which affect the feeding process^(34)^. According to a systematic review conducted in sub-Saharan countries, including Ethiopia, one in five children (19⋅8 %) had significant difficulties, and one in ten (9⋅5 %) had a specific mental disorder^(35)^. Another study found that about 23 % of children in Ethiopia have mental health problems^(36)^. Therefore, understanding the role of mental health problems in children and mothers/caregivers in the use of specific child feeding practices is one of the steps in tailoring interventions to improve feeding interaction and malnutrition prevention programmes^(28)^.

It is currently not known how prevalent these controlling feeding practices are, how frequently they are employed or whether their use differs by the various child and caregivers' characteristics in Ethiopia. Understanding the local context is imperative to explain the dynamics of the child feeding process and its factors. Hence, this study aims to fill the existing literature gap and examine the association between caregivers' feeding practices and caregiver and child characteristics among preschool children in Addis Ababa.

## Materials and methods

### Participants and procedures

This school-based cross-sectional study was employed among 542 caregivers of children aged between three and six attending preschools in Addis Ababa, the capital city of Ethiopia. The sample size was determined using WINPEPI software version 11.65 by taking standard deviation from a previous study^(37)^. The calculated sample size was 325, which was later changed to 542 samples after the addition of design effect and non-response rate. In addition, the study was 90 % adequately powered.

We used a multi-stage sampling technique to obtain a representative sample of study participants. The study was conducted on all children attending the selected preschools in Addis Ababa in 2018/2019 and their parents/caregivers. To select the preschools, we initially stratified sub-cities where the preschools are divided into three strata using socioeconomic status indicators. Secondly, we randomly selected one sub-city from each stratum. Thirdly, we used a probability proportional to size sampling to select four preschools from each sub-city. Finally, we selected study participants by randomly using preschool registers from each grade level.

Parents/caregivers of the randomly selected children were recruited by an invitation to participate in the study written by the preschool teachers in the children's communication notebooks. Informed consent was obtained after explaining the primary purpose of the study. Finally, the parents/caregivers completed an anonymous interview-based questionnaire, and the children's anthropometric measurements were taken the next day.

### Ethical standards disclosure

This study was conducted according to the guidelines laid down in the Declaration of Helsinki, and all procedures involving research study participants were approved by the ethical review board of Addis Ababa University under the project (No. 0011). Written informed consent was obtained from all caregivers.

### Measurements

#### Outcome variables

##### Feeding practices

*Caregiver's feeding practices were assessed through the CFQ*^(2)^: Three subscales were used to measure feeding practices: (i) restriction (eight items); (ii) pressure to eat (four items); (iii) monitoring (three items). The responses to restriction and pressure to eat practices were categorised on a five-point Likert scale (1 disagree, 2 slightly disagree, 3 neutral, 4 slightly agree, 5 agree). The responses to monitoring practices were also categorised on a five-point Likert scale (1 never, 2 rarely, 3 sometimes, 4 often, 5 always). Each scale was generated by calculating the mean of the items and was subsequently expressed as mean and standard deviations. A higher scale score represents more frequent use of a feeding strategy.

The CFQ is generally used to measure caregivers' beliefs, attitudes and practices regarding child feeding. It is reported to have good internal consistency, with a Cronbach's alpha value ranging from 0⋅70 to 0⋅92^(2)^. Similarly, this study also found a good internal consistency regarding restriction (*α* 0⋅74), pressure to eat (*α* 0⋅71) and monitoring (*α* 0⋅84) feeding practice scales. In the present study, the CFQ was first translated by a qualified researcher into an Amharic version and then back-translated into English by a different researcher to ensure the quality of the translation and maintain consistency in the questionnaire. The researcher was independent of the study to ensure the accuracy of the Amharic version. Face validity was conducted for the thirty-one items of the CFQ^(2)^ and concern about child underweight scale (three items) in the Preschooler Feeding Questionnaire (PFQ)^(38)^. Two focus group discussions with twenty parents, ten parents per focus group, were recruited from two preschools that were not included in the study. The focus group discussion was conducted to clarify wording and enable the target audience to understand and answer the questions as intended. Some of the discussion points included how to best structure the items in a way that could be understood in the context of the country without missing the main intention of the questions. Participants were also asked to suggest ideas on how to improve the wording of items that were perceived as vague or unclear, and constructive feedback from parents in this step was used to refine the questionnaire. During the focus group discussion, three parents were unsure about the meaning of the equivalent Amharic terminologies for perceived child weight, item 1: ‘Your child during the first year of life’ and for concern about child overweight: ‘How concerned are you about your child having to diet to maintain a desirable weight?’ In addition, based on the constructive feedback from parents, we decided to clarify and rephrase words, such as ‘high-fat foods’ and ‘junk food’, from the restriction and monitoring scale into more widely accessible and contextually acceptable phrases when asking about food types. The remaining items were kept in their original form since there were no problems in understanding them. To test the whole questionnaire, a pilot study was conducted on fifty preschool children and their parents in three different preschools that were not included in the study.

#### Independent variables

##### Perceived feeding responsibility, perceived caregiver weight and child weight, and concern about child overweight/underweight

The CFQ was also used to assess perceived feeding responsibility, perceived caregiver weight and child weight, and concern about child overweight. The perceived feeding responsibility scale has three items and measures caregivers' perceptions of their child's feeding responsibility. It is answered using a five-point Likert scale (1 never, 2 seldom, 3 half of the time, 4 most of the time, 5 always).

The perceived caregiver and child weight scales have four and three items, respectively, and measure caregivers' perceptions of their own and their child's weight status history. It is answered using a five-point Likert scale (1 markedly underweight, 2 underweight, 3 normal, 4 overweight, 5 markedly overweight). The concern about the child overweight scale has three items which assess caregivers' concerns about the child's risk of being overweight and is answered using a five-point Likert scale (1 unconcerned, 2 a little concerned, 3 concerned, 4 fairly concerned, 5 very concerned). The concern about child underweight scale assesses caregivers' concerns about the child's risk of being underweight and is answered using a five-point Likert frequency scale (1 never, 2 rarely, 3 sometimes, 4 often, 5 always), which was taken from the PFQ^(38)^.

Each scale was generated by calculating the mean of the items and was subsequently expressed as mean and standard deviations. A higher scale score represents greater concern, and higher levels of perception represent a higher weight status. The present study has found a good internal consistency regarding perceived feeding responsibility (*α* 0⋅83), perceived parent weight (*α* 0⋅76) and perceived child weight (*α* 0⋅89). In addition, concern about child overweight and concern about child underweight have Cronbach's alpha values of 0⋅61 and 0⋅64, respectively.

##### Caregivers’ depressive symptoms

Caregivers' depressive symptoms were measured using the Patient Health Questionnaire-9 (PHQ-9), a nine-item self-report measure used to screen depressive disorders, and that has been validated in Ethiopia^(39)^. The items ask how frequently caregivers have been bothered by depressive symptoms over the past 2 weeks, with response categories of 0 ‘not at all’, 1 ‘several days’, 2 ‘more than half the days’ and 3 ‘nearly every day’. Caregivers' depression status was assessed using the standard total PHQ-9 score, which was then divided into three categories: (i) no depression; (ii) mild depression; (iii) moderate depression.

##### Children's mental health status

The children's mental health status was assessed using the Strengths and Difficulties Questionnaire (SDQ), specifically, the parent or caregiver report version (SDQ-P)^(40)^. This measures both mental health difficulties and competencies, using the categorisation of total difficulty scores found by adding the scores from all the scales except the prosocial scale. An authorised Amharic version of the SDQ is available^(41)^. It has also been shown to have a good construct and convergent validity in the Ethiopian context (unpublished results). The children's mental health status was categorised using the standard SDQ parent/caregiver version categorisation of total difficulty scores. The resultant score was divided into four groups: (i) close to average; (ii) slightly raised; (iii) high; (iv) very high.

##### Anthropometric measurements

Anthropometric measurements were taken to compute the children's BMI. Weight was measured to the nearest 0⋅1 kg using a portable electronic scale (Seca), and height was measured in the standing position to the nearest 0⋅1 cm using a portable locally made stadiometer. We conducted an anthropometric measurement (weight and height) standardisation exercise among selected children and calculated the intra- and inter-observer technical errors of measurement (TEM). The intra-observer TEM was 0⋅19 and 0⋅12 for height and weight, respectively, while the inter-observer TEM was 0⋅21 for both height and weight. The coefficient of reliability was 97⋅5 %. All findings were found to be within an acceptable range.

The World Health Organization's (WHO) 2007 growth reference was used as a standard reference for classifying preschool children's BMI using the WHO's Anthro Plus software, version 1.0.21. Children's weight status was classified using the WHO's 2007 growth reference for BMI-for-age cut-offs. Children aged 5 years and above with BMI- for-age z-score (BAZ) less than −3 were classified as severely underweight, and children between −3 and −2 as underweight, between −2 and +1 as normal, between +1 and +2 as overweight, and greater than +2 as obese^(42)^. Children aged below 5 years with BAZ less than −3 were classified as severely underweight, and children between −3 and −2 as underweight, between −2 and +1 as normal, between +1 and +2 as at risk of overweight, between +2 and +3 as overweight, and greater than +3 as obese^(43)^.

##### Demographic and socioeconomic characteristics

Demographic characteristics, including the child's sex, age (in completed years) and the caregivers' education level, were obtained. Socioeconomic status was assessed through the ownership of household assets and housing conditions-related variables.

The socioeconomic status of the study households was determined using the principal component analysis. Household asset ownership and housing condition-related variables were used in the analysis to categorise households into wealth quintiles, ranging from the poorest to the richest.

### Statistical analysis

We used the statistical software Stata version 15.0 for analysis. Descriptive data were presented using frequency, percentage and mean (sd). All the analyses performed were pre-specified and based on the study objective.

Three separate multiple linear regression models while adjusting for each beta coefficient were run to determine the associations between each variable of caregiver and child characteristics, such as child's age, child's sex, child's BMI *z* score, child's mental health status, caregiver's depression status, caregiver's perceived feeding responsibility, perceived caregiver weight, child weight status, concern about child over/underweight, caregivers' education level and socioeconomic status and each scale of caregivers' feeding practices. We used multiple linear regression because this model provides a way of adjusting for (or accounting for) potentially confounding variables that have been included in the model, which further assesses each independent variable and the outcome, holding all other variables constant. Statistical significance was set at a value of *P* < 0⋅05.

## Results

We approached a total of 542 caregivers, of which 525 participated in the study, resulting in a response rate of 96⋅8 %. A total of seventeen caregiver–child dyads were not included in the study because fourteen of the caregivers did not have adequate time to be interviewed, and three of the children were unwilling to remove their shoes or other clothing during the anthropometric measurements.

The sociodemographic characteristics of caregiver–child dyads are presented in Table 1. Most of the respondents (92⋅2 %) were the mothers of the index children, and 63⋅7 % of the respondents had secondary education or higher. The mean age of the children was 4⋅5 years (sd 0⋅04). We found that 76⋅9 % (404) of the children had normal BMI-for-age scores. However, 8⋅0 % (forty-two) of the children were overweight, and 2⋅9 % (fifteen) were obese. Our findings also showed that 5⋅9 % (thirty-one) of the children were underweight. The majority of the caregivers (57⋅1 %) had no symptoms of depression, while 15⋅2 % (eighty) of the caregivers had moderate depression symptoms. We also found that the majority of the children (60⋅3 %) scored close to average, while 15⋅4 % (eighty-one) scored very high on the SDQ-P total difficulty score (see Table 1).

**Table 1.** Characteristics of the preschool children and their caregivers

Variables	*N*	%	Mean values	sd
Child's age (in year)			4⋅5	0⋅04
Child's sex				
Male	247	47		
Female	278	53		
Caregivers' relation to the child				
Mother	484	92⋅2		
Father	14	2⋅7		
Grandmother	10	1⋅9		
Other	17	3⋅2		
Caregivers' educational level				
No formal education	59	11⋅2		
Primary education	131	24⋅9		
Secondary education	183	34⋅8		
Technical school and above	152	28⋅9		
Wealth index				
Poorest	105	20⋅1		
Poor	104	19⋅9		
Medium	104	19⋅9		
Wealthy	104	19⋅9		
Wealthiest	104	19⋅9		
Type of school				
Private/missionary/church	389	74⋅1		
Government/public	136	25⋅9		
Child's BMI				
Underweight	31	5⋅9		
Normal weight	404	76⋅9		
At risk of overweight	33	6⋅3		
Overweight	42	8⋅0		
Obese	15	2⋅9		
Caregivers' depression status				
No depression	300	57⋅1		
Mild depression	145	27⋅6		
Moderate depression	80	15⋅2		
Child's mental health status				
Close to average	317	60⋅3		
Slightly raised	78	14⋅8		
High	49	9⋅3		
Very high	81	15⋅4		

Table 2 shows the mean scores of the seven-scale CFQ and concern about child underweight scale taken from the PFQ. For the feeding practices, monitoring had the highest mean score of 4⋅13 (sd 1⋅02), indicating that caregivers do keep track of what their child eats most of the time, while restriction had the lowest score of 3⋅47 (sd 0⋅91), indicating that caregivers slightly agreed with the practice of restricting the intake of some types of food by their child. In addition, perceived feeding responsibility had the highest score out of all the scales (mean 4⋅47, sd 0⋅80), indicating that parents considered themselves responsible for feeding their child most of the time. Regarding the perceived caregiver's weight factor (mean 3⋅01, sd 0⋅47) and the perceived child weight factor (mean 3⋅05, sd 0⋅56), caregivers reported normal weight from childhood up to the present moment for themselves and their child. These scores also likely reflect that 76⋅9 % of the sample of children have a healthy weight status. In addition, the internal reliability (Cronbach's alpha) for each subscale ranged from 0⋅61 to 0⋅89.

**Table 2.** Mean scale scores from the CFQ and one scale from the PFQ in Addis Ababa, Ethiopia

Variables	Mean values	sd	Cronbach's alpha
Perceived feeding responsibility	4⋅47	0⋅80	0⋅83
Perceived parent weight	3⋅01	0⋅47	0⋅76
Perceived child weight	3⋅05	0⋅56	0⋅89
Concern about child overweight	2⋅92	1⋅16	0⋅61
Concern about child underweight^a^	2⋅42	1⋅35	0⋅64
Restriction	3⋅47	0⋅91	0⋅74
Pressure to eat	4⋅02	0⋅99	0⋅71
Monitoring	4⋅13	1⋅02	0⋅84

a Concern about child underweight is taken from a PFQ.

Table 3 presents a separate multivariable linear regression model fitted to investigate the association between caregiver and child characteristics and each scale of caregivers' feeding practices. These characteristics account for sociodemographic characteristics, caregivers' depression status, child's mental health status, caregivers' perceived feeding responsibility, caregivers' perceptions of their own and their child's weight, and concern about child underweight and overweight.

**Table 3.** Multivariable linear regression analysis showing the association between three scales of feeding practice with parent and child characteristics in Addis Ababa, Ethiopia

Adjusted^a^	Parental feeding practice		
	Restriction *β* (95 % confidence intervals)	Pressure to eat *β* (95 % confidence intervals)	Monitoring *β* (95 % confidence intervals)
Parent characteristics			
Parent educational level			
Primary education	0⋅12 (−0⋅16, 0⋅41)	−0⋅07 (−0⋅37, 0⋅23)	0⋅27 (−0⋅01,0⋅56)
Secondary education	0⋅17 (−0⋅10, 0⋅45)	−0⋅14 (−0⋅44, 0⋅14)	0⋅41 (0⋅13, 0⋅69)**
Technical school and above	0⋅21 (−0⋅07, 0⋅50)	−0⋅12 (−0⋅43, 0⋅17)	0⋅27 (−0⋅01, 0⋅56)
Wealth index			
Poor	−0⋅02 (−0⋅27, 0⋅21)	−0⋅21 (−0⋅47, 0⋅04)	0⋅17 (−0⋅07, 0⋅42)
Medium	0⋅03 (−0⋅20, 0⋅28)	0⋅01 (−0⋅25, 0⋅26)	0⋅07 (−0⋅19, 0⋅30)
Wealthy	−0⋅10 (−0⋅36, 0⋅14)	0⋅06 (−0⋅20, 0⋅32)	0⋅11 (−0⋅13, 0⋅36)
Wealthiest	−0⋅02 (−0⋅27, 0⋅22)	−0⋅01 (−0⋅26, 0⋅25)	0⋅17 (−0⋅07, 0⋅42)
Parent depression status			
Mild depression	0⋅11 (−0⋅07, 0⋅29)	0⋅02 (−0⋅17, 0⋅22)	0⋅08 (−0⋅10, 0⋅27)
Moderate depression	0⋅23 (−0⋅01, 0⋅47)*	−0⋅23 (−0⋅48, 0⋅02)	−0⋅03 (−0⋅27, 0⋅21)
Perceived feeding responsibility	0⋅20 (0⋅10, 0⋅30)**	0⋅25 (0⋅15, 0⋅35)**	0⋅47 (0⋅03, 0⋅57)**
Perceived parent weight	−0⋅02 (−0⋅19, 0⋅13)	−0⋅09 (−0⋅27, 0⋅07)	−0⋅11 (−0⋅27, 0⋅05)
Perceived child weight	−0⋅01 (−0⋅13, 0⋅15)	0⋅07 (−0⋅08, 0⋅22)	0⋅05 (−0⋅09, 0⋅20)
Concern about child underweight	0⋅01 (−0⋅04, 0⋅07)	0⋅15 (0⋅08, 0⋅21)**	−0⋅06 (−0⋅12, −0⋅01)*
Concern about child overweight	0⋅11 (0⋅04, 0⋅18)**	−0⋅10 (−0⋅17,−0⋅02)**	0⋅15 (0⋅07, 0⋅22)**
Child characteristics			
Age in years	0⋅04 (−0⋅01, 0⋅15)	0⋅05 (−0⋅03, 0⋅13)	0⋅08 (0⋅01, 0⋅16)*
Sex: female	0⋅04 (−0⋅11, 0⋅20)	0⋅16 (−0⋅01, 0⋅32)	−0⋅11 (−0⋅27, 0⋅04)
Child's nutritional status			
BMI-for-age score	−0⋅01(−0⋅07, 0⋅05)	0⋅04 (−0⋅10, 0⋅02)	0⋅02 (−0⋅09, 0⋅03)
Child's mental health status			
Slightly raised	0⋅12 (−0⋅10, 0⋅34)	−0⋅01 (−0⋅24, 0⋅23)	−0⋅03 (−0⋅25, 0⋅20)
High	0⋅01 (−0⋅26, 0⋅28)	−0⋅12 (−0⋅42, 0⋅16)	0⋅03 (−0⋅24, 0⋅31)
Very high	0⋅12 (−0⋅11, 0⋅35)	−0⋅13 (−0⋅37, 0⋅11)	−0⋅08 (−0⋅31, 0⋅14)

Reference groups: sex, male; educational level, no formal education; wealth index, very poor; depression status, no depression; total difficulty score, normal.

a Each model adjusted for caregiver's education status, caregiver's wealth index, caregiver's depression status, perceived feeding responsibility, perceived caregiver's weight, perceived child weight, concern about child underweight and concern about child overweight, child's age in years, child's sex, child's mental status and child's BMI-for-age.

* *P* < 0⋅05, **P < 0⋅01.

### Food restriction, caregiver and child characteristics

We found a significant positive association between perceived feeding responsibility (*β* 0⋅20, *P* < 0⋅001), concern about child overweight (*β* 0⋅11, *P* < 0⋅001), caregivers' depression status (no depression as a reference group) (*β* 0⋅23, *P* 0⋅05) and caregivers' food restriction practices (see Table 3).

### Pressure to eat, caregiver and child characteristics

We found a significant positive association between perceived feeding responsibility (*β* 0⋅25, *P* < 0⋅001), concern about child underweight (*β* 0⋅15, *P* < 0⋅001) and caregivers' pressure to eat practices. Moreover, a significant negative association was found between concern about child overweight and pressure to eat practice (*β* −0⋅10, *P* < 0⋅001), indicating that on average, those caregivers who have a concern about their child being overweight have 0⋅10 lower practice of pressure to eat (see Table 3).

### Monitoring, caregiver and child characteristics

We found a significant positive association between child's age (*β* 0⋅08, *P* < 0⋅05), caregivers' education (*β* 0⋅41, *P*  < 0⋅001), perceived feeding responsibility (*β* 0⋅47, *P* < 0⋅001), concern about child overweight (*β* 0⋅15, *P* < 0⋅001) and caregivers' monitoring practices. In addition, a significant negative association was found between concern about child underweight and caregivers' monitoring practices (*β* −0⋅06, *P* < 0⋅05), indicating that on average, those caregivers that have a concern about their child being underweight have 0⋅06 lower practice of monitoring feeding practice (see Table 3).

## Discussion

The main aim of this study was to examine whether caregiver and child characteristics are associated with feeding practices among parents/caregivers of preschool children in Ethiopia. This study shows the average use of feeding practices and their association with various characteristics in understudied Ethiopian populations.

Caregivers concerned about child's weight have a significant association with their feeding practices. For instance, concern about child overweight has a significant positive association with caregivers' food restriction and monitoring practices, while it has a significant negative association with pressure to eat. This finding was in line with a study conducted in the United States that found that caregivers concerned about their child becoming/being overweight were less likely to practice pressure to eat and more likely to restrict the consumption of unhealthy foods^(44)^. This may be because there is now a better understanding of the dangers of being overweight and its different psychological effects^(25)^. This is further supported by the finding from a recent study done in one of the growing cities of Ethiopia, where a high prevalence of overweight/obesity (13⋅8 %)^(45)^ was recorded compared to the previous national study^(20)^. However, some studies found that parental/caregivers' concern about child weight is not significantly associated with monitoring^(3,14)^. Also, in most sub-Saharan countries including Ethiopia, a significant decline in diet quality and a high intake of unhealthy food items have been observed^(46)^. Consequently, caregivers in Addis Ababa are now concerned and understand that these ‘unhealthy foods’ are not good and are unsafe for their children^(26)^, making it inevitable that caregivers will apply more restrictive feeding practices. Pressure to eat is mainly practiced to sustain adequate food consumption. It is associated with children eating less and having a lower weight status^(8)^. Therefore, concern about their child being or becoming underweight instead of being overweight might be the main reason caregivers pressurise their child to eat. This can further be explained by the finding in our study, where concern about child underweight has a significant positive association with caregivers' pressure to eat. This is similarly reported in an Australian study, where mothers concerned about child underweight showed a positive association with pressure to eat practice^(3)^.

Experimental studies have found that restricting access to certain foods has been related to an increase in children's preferences for those foods and has been positively associated with BMI^(15,16)^. In the meantime, parental use of pressure to eat has been shown to cause negative affective reactions to the foods and an association with low weight in children^(14,15)^. However, studies have also found that caregivers/parents of children with a higher BMI tend to be more restrictive^(10,47)^, whereas caregivers/parents of children with a lower BMI tend to use pressure to eat more often^(47)^. Thus, some studies have explored this complex relationship and have found a bidirectional effect between caregivers feeding practice and child weight status^(47,48)^, indicating that caregivers may be both responding to and influencing their children's weight. Nevertheless, this study did not find any association between child BMI and caregivers' feeding practices in our multivariable analysis. This finding was similar to an Australian study, which found that child BMI was not a significant predictor for maternal use of pressure to eat and restriction feeding practice^(3)^. In addition, studies have found that most caregivers misperceive/misclassify the weight of their child^(49,50)^. This might suggest that caregivers are inclined to use these feeding strategies in response to their concern about their child's weight instead of the child's actual BMI measurements^(3)^. By contrast, a prospective study conducted in the Netherlands found that children with lower BMI scores predicted higher levels of parental pressure to eat, whereas parents tended to practice restriction in response to relatively high child BMI^(47)^.

Caregiver's perceived feeding responsibility is associated with caregiver's food restriction, pressure to eat and monitoring. This indicates that caregivers in our study sample who practice any of the feeding practices consider themselves responsible for feeding their child most of the time. Similarly, another study found that parents who perceived themselves as more responsible for their child's feeding had an association with pressure to eat and restrictive practices^(51)^. However, it was also found that feeding responsibility was related to monitoring practice, higher teaching of their children about nutrition, higher modelling of healthy eating habits and higher encouragement of balanced and varied diets^(52)^. Perhaps, caregivers in the present study felt responsible for feeding their children but lacked the differentiation between responsive feeding practices that support the development of healthier food intake and controlling feeding practices that are associated with poorer diets. Therefore, it is necessary to reinforce the use of responsive feeding practices through nutritional education in interventions focused on feeding interaction.

Caregivers' practice of food restriction is likely to affect children's natural signals of hunger and satiety cues^(53)^. Caregivers' food restriction has been shown to mediate a positive relationship between caregivers/mothers with depressive symptoms and childhood obesity^(54)^. This can further be explained in our study, which showed that caregivers' depression status is positively associated with food restriction. However, findings from Western countries are mixed. For instance, an Australian study reported that high levels of mothers' depression are associated with the use of restriction (food as a reward/instrumental feeding)^(55)^. By contrast, a US study showed no relation between symptoms of depression and observations of the maternal practice of food restriction^(56)^.

On the other hand, we found no association between children's mental health status (total difficulty score) and caregivers' feeding practices. This might be because parents/caregivers with a child who is more irritable and in distress may be more likely to engage in other types of feeding practice, such as permissive practices, where parents/caregivers allow their children to make their own decision about food and set no boundaries to avoid additional stress^(57)^. However, a US study found that parents are more likely to use pressure to eat on children with some negative temperamental traits^(58)^. Negative affectivity may result in difficult communication between children and parents, which could influence feeding practices and the child's eating behaviour^(59)^. Therefore, further research should be conducted on the association between children's mental health status and caregivers' feeding practices because they are important for preventing unhealthy eating behaviours and may be especially harmful to children with mental health difficulties.

Another pertinent finding in this study is the association between sociodemographic characteristics and caregivers' feeding practice. Caregivers' educational level was found to have a significant association with monitoring feeding practice. This finding was similar to a study conducted in Belgium, where mothers with high levels of education abstained from sweets in the presence of their children more often than less educated mothers^(60)^. This may suggest that mothers who have higher educational levels tend to be responsive, feed their children healthier, introduce more diverse products at an early age and value a balanced diet above cost in their food choices^(61)^. By contrast, a Brazilian study found that lower maternal/caregiver education did not show any association with lower monitoring practice^(25)^.

Moreover, our study found that, as the children got older, there is an increased use of monitoring feeding practice. This may be because as the children get older, they are more likely to have more independence and be more exposed and vulnerable to unhealthy food choices^(62)^. This may increase caregivers' monitoring of food choices to manipulate their children's eating behaviour and encourage healthy food consumption.

This study has significant strengths, including (i) an adequate sample size with the use of probability sampling techniques which help to generalise and (ii) the use of standardisation protocols for anthropometric measurements, which help obtain accurate and precise anthropometric measurements and reduce errors. There are, however, limitations to our study that should be considered. First, the study's cross-sectional nature hinders the inference of causal conclusions of child and caregiver factors and caregivers' feeding practices, since it is unclear whether feeding practices are reactive to these factors or whether some caregiver/child factors result from feeding practices. Future studies can utilise longitudinal research designs to assess these complex relationships. Secondly, the subjective introduction of bias cannot be ruled out, since the measurements were based on the caregivers' self-reporting rather than on direct observation of caregiver feeding practices. Although there are many advantages to employing observations and laboratory tests instead of questionnaires, these techniques are not cost-effective, especially in population studies such as the present one. Future research may prefer to use observational methodology in naturalistic settings, enhancing our understanding of the different caregiver feeding practices used. Important factors, such as ethnicity, parent nutritional knowledge, childbirth weight, parent weight and infant feeding practices, are worth including multivariable regression analysis in future studies. The other drawback is that the CFQ has not been validated in this study area, even though the CFQ has been validated in similar settings^(37)^, and the effect has been minimised by performing face validity and internal reliability (Cronbach's alpha) tests on a sample of caregivers before using the measurement scale in this study. This might jeopardise the tool's ability to assess what it is intended to measure accurately. It might also be one of the reasons for the observed low Cronbach's alpha value in concern about child overweight and underweight scale. However, Cronbach's alpha is quite sensitive to the number of items in the scale, and it is common to find scales with low Cronbach's alpha values when the items are less than ten^( )^. Therefore, future studies should further check the applicability and sensitivity to the cultural differences of the above questionnaire.

### 

#### Conclusions

This study is the first to show the association between caregiver and child characteristics and caregiver feeding practices in developing countries such as Ethiopia. Caregivers who had higher levels of perceived feeding responsibility, who were more concerned about their child being overweight and who had higher scores for depressive symptoms were associated with food restriction practice. Caregivers who were less concerned about their child being overweight, who had higher levels of perceived feeding responsibility and who were more concerned about their child being underweight were associated with pressure to eat practice. Caregivers who had higher education and higher levels of perceived feeding responsibility, who were more concerned about their child being overweight and who were less concerned about child underweight were associated with monitoring feeding practice. In addition, as the children have gotten older, there is an increased use of monitoring feeding practice.

In Ethiopia, it is essential to include a responsive feeding component in national nutritional programmes to improve preschool children's nutritional status. In addition, reinforcing the use of responsive feeding practices through nutrition education provided by health care providers will help parents and other caregivers be aware of appropriate feeding practices.

## References

[1] Liu YH & Stein MT (2012) Feeding behaviour of infants and young children and its impact on child psychosocial and emotional development. In *Encyclopedia on* Early Childhood Development. Montreal: Centre of Excellence for Early Childhood Development Strategic Knowledge Cluster on Early Child Development.

[2] Birch LL, Fisher JO, Grimm-Thomas K, (2001) Confirmatory factor analysis of the Child Feeding Questionnaire: a measure of parental attitudes, beliefs and practices about child feeding and obesity proneness. Appetite 36, 201–210. 11358344 10.1006/appe.2001.0398

[3] Gregory JE, Paxton SJ & Brozovic AM (2010) Pressure to eat and restriction are associated with child eating behaviours and maternal concern about child weight, but not child body mass index, in 2-to 4-year-old children. Appetite 54, 550–556. 20219609 10.1016/j.appet.2010.02.013

[4] Ventura AK & Birch LL (2008) Does parenting affect children's eating and weight status? Int J Behav Nutr Phys Act 5, 15. 18346282 10.1186/1479-5868-5-15 PMC2276506

[5] Rimm-Kaufman SE & Pianta RC (2000) An ecological perspective on the transition to kindergarten: a theoretical framework to guide empirical research. J Appl Dev Psychol 21, 491–511.

[6] Ashcroft J, Semmler C, Carnell S, (2008) Continuity and stability of eating behaviour traits in children. Eur J Clin Nutr 62, 985–990. 17684526 10.1038/sj.ejcn.1602855

[7] Mitchell GL, Farrow C, Haycraft E, (2013) Parental influences on children's eating behaviour and characteristics of successful parent-focussed interventions. Appetite 60, 85–94. 23017468 10.1016/j.appet.2012.09.014

[8] Jansen PW, Roza SJ, Jaddoe VW, (2012) Children's eating behavior, feeding practices of parents and weight problems in early childhood: results from the population-based Generation R Study. Int J Behav Nutr Phys Act 9, 130. 23110748 10.1186/1479-5868-9-130 PMC3543222

[9] Wan Abdul Manan W, Norazawati A & Lee Y (2012) Overweight and obesity among Malay primary school children in Kota Bharu, Kelantan: parental beliefs, attitudes and child feeding practices. Malays J Nutr 18(1), 27–36. 23713227

[10] Gregory JE, Paxton SJ & Brozovic AM (2010) Maternal feeding practices, child eating behaviour and body mass index in preschool-aged children: a prospective analysis. Int J Behav Nutr Phys Act 7, 55. 20579397 10.1186/1479-5868-7-55 PMC2907299

[11] Birch LL & Fisher JO (2000) Mothers’ child-feeding practices influence daughters’ eating and weight. Am J Clin Nutr 71, 1054–1061. 10799366 10.1093/ajcn/71.5.1054 PMC2530928

[12] Bante H, Elliott M, Harrod A, (2008) The use of inappropriate feeding practices by rural parents and their effect on preschoolers’ fruit and vegetable preferences and intake. J Nutr Educ Behav 40, 28–33. 18174101 10.1016/j.jneb.2007.02.007

[13] Loth KA (2016) Associations between food restriction and pressure-to-eat parenting practices and dietary intake in children: a selective review of the recent literature. Curr Nutr Rep 5, 61–67.

[14] Webber L, Hill C, Cooke L, (2010) Associations between child weight and maternal feeding styles are mediated by maternal perceptions and concerns. Eur J Clin Nutr 64, 259–265. 20087383 10.1038/ejcn.2009.146 PMC2875105

[15] Tschann JM, Martinez SM, Penilla C, (2015) Parental feeding practices and child weight status in Mexican American families: a longitudinal analysis. Int J Behav Nutr Phys Act 12, 66. 25986057 10.1186/s12966-015-0224-2 PMC4453102

[16] Hughes SO, Power TG, O'Connor TM, (2016) Maternal feeding styles and food parenting practices as predictors of longitudinal changes in weight status in Hispanic preschoolers from low-income families. Int J Obes 2016, 7201082. 10.1155/2016/7201082 PMC4939194 27429801

[17] Abdullah A (2015) The double burden of undernutrition and overnutrition in developing countries: an update. Curr Obes Rep 4, 337–349. 26627492 10.1007/s13679-015-0170-y

[18] Tariku A (2016) Effect of Feeding Style on Intake of Complementary Foods Appetite and Nutritional Status of Infants Aged 9–11 Months in West Gojam Ethiopia. (Doctoral dissertation, Addis Ababa University).

[19] Berhane HY, Jirström M, Abdelmenan S, (2020) Social stratification, diet diversity and malnutrition among preschoolers: a survey of Addis Ababa, Ethiopia. Nutrients 12, 712. 10.3390/nu12030712 PMC7146462 32156006

[20] CSA (2016) Ethiopia Demographic Health Survey. Addis Ababa, Ethiopia Calverton, Maryland, USA. Available at http://dhsprogram.com/publications/publication-FR328-DHS-Final-Reports.cfm (accessed 25 February 2020).

[21] Gebru NW, Gebreyesus SH, Yirgu R, (2020) The relationship between caregivers’ feeding practices and children's eating behaviours among preschool children in Ethiopia. Appetite 157, 104992. 33049339 10.1016/j.appet.2020.104992

[22] Abebe Z, Haki GD & Baye K (2017) Child feeding style is associated with food intake and linear growth in rural Ethiopia. Appetite 116, 132–138. 28461197 10.1016/j.appet.2017.04.033

[23] Wondafrash M, Amsalu T & Woldie M (2012) Feeding styles of caregivers of children 6–23 months of age in Derashe special district, Southern Ethiopia. BMC Public Health 12, 235. 22439749 10.1186/1471-2458-12-235 PMC3326699

[24] Savage JS, Fisher JO & Birch LL (2007) Parental influence on eating behavior: conception to adolescence. J Law Med Ethics 35, 22–34. 17341215 10.1111/j.1748-720X.2007.00111.x PMC2531152

[25] Warkentin S, Mais LA, De Oliveira MDRD, (2018) Relationships between parent feeding behaviors and parent and child characteristics in Brazilian preschoolers: a cross-sectional study. BMC Public Health 18, 704. 29880038 10.1186/s12889-018-5593-4 PMC5992628

[26] Berhane HY, Ekström E-C, Jirström M, (2018) What influences urban mothers’ decisions on what to feed their children aged under five – the case of Addis Ababa, Ethiopia. Nutrients 10, 1142. 10.3390/nu10091142 PMC6164347 30135354

[27] McLearn KT, Minkovitz CS, Strobino DM, (2006) The timing of maternal depressive symptoms and mothers’ parenting practices with young children: implications for pediatric practice. Pediatrics 118, e174–e182. 16818531 10.1542/peds.2005-1551

[28] McPhie S, Skouteris H, Daniels L, (2014) Maternal correlates of maternal child feeding practices: a systematic review. Matern Child Nutr 10, 18–43. 22973806 10.1111/j.1740-8709.2012.00452.x PMC6860315

[29] Haycraft E (2020) Mental health symptoms are related to mothers’ use of controlling and responsive child feeding practices: a replication and extension study. Appetite 147, 104523. 31756410 10.1016/j.appet.2019.104523

[30] Blissett J, Haycraft E & Farrow C (2010) Inducing preschool children's emotional eating: relations with parental feeding practices. Am J Clin Nutr 92, 359–365. 20534744 10.3945/ajcn.2010.29375

[31] Fantahun A, Cherie A & Deribe L (2018) Prevalence and factors associated with postpartum depression among mothers attending public health centers of Addis Ababa, Ethiopia, 2016. Clin Pract Epidemiol Ment Health 14, 196. 30258485 10.2174/1745017901814010196 PMC6131316

[32] Anato A, Baye K, Tafese Z, (2019) Maternal depression is associated with child undernutrition: a cross-sectional study in Ethiopia. Matern Child Nutr 16(3), e12934. 31833231 10.1111/mcn.12934 PMC7296785

[33] Klein AM, Otto Y, Fuchs S, (2015) A prospective study of behavioral and emotional symptoms in preschoolers. Eur Child Adolesc Psychiatry 24, 291–299. 24972693 10.1007/s00787-014-0575-2

[34] Zucker N, Copeland W, Franz L, (2015) Psychological and psychosocial impairment in preschoolers with selective eating. Pediatrics 136, e582–e590. 26240213 10.1542/peds.2014-2386 PMC4552088

[35] Cortina MA, Sodha A, Fazel M, (2012) Prevalence of child mental health problems in sub-Saharan Africa: a systematic review. Arch Pediatr Adolesc Med 166, 276–281. 22393184 10.1001/archpediatrics.2011.592

[36] WHO (2015) Introducing School Mental Health in Ethiopia. Geneva, Switzerland: The Department of Mental Health and Substance Abuse, Mental Health Innovation Network.

[37] Do LM, Eriksson B, Tran TK, (2015) Feeding of preschool children in Vietnam: a study of parents’ practices and associated factors. BMC Nutr 1, 16.

[38] Baughcum AE, Powers SW, Johnson SB, (2001) Maternal feeding practices and beliefs and their relationships to overweight in early childhood. J Dev Behav Pediatr 22, 391–408. 11773804 10.1097/00004703-200112000-00007

[39] Gelaye B, Williams MA, Lemma S, (2013) Validity of the Patient Health Questionnaire-9 for depression screening and diagnosis in East Africa. Psychiatry Res 210, 653–661. 23972787 10.1016/j.psychres.2013.07.015 PMC3818385

[40] Goodman R (1997) The Strengths and Difficulties Questionnaire: a research note. J Child Psychol Psychiatr 38, 581–586. 10.1111/j.1469-7610.1997.tb01545.x 9255702

[41] Goodman R (2002) 2013 http://www.sdqinfo.com.

[42] Van den Broeck J, Willie D & Younger N (2009) The World Health Organization child growth standards: expected implications for clinical and epidemiological research. Eur J Pediatr 168, 247–251. 18670787 10.1007/s00431-008-0796-9

[43] Organization WH (2006) WHO Child Growth Standards: Length/Height-for-Age, Weight-for-Age, Weight-for-Length, Weight-for-Height and Body Mass index-for-Age: Methods and Development. World Health Organization.

[44] May AL, Donohue M, Scanlon KS, (2007) Child-feeding strategies are associated with maternal concern about children becoming overweight, but not children's weight status. J Am Diet Assoc 107, 1167–1174. 17604746 10.1016/j.jada.2007.04.009

[45] Sorrie MB, Yesuf ME & GebreMichael TG (2017) Overweight/obesity and associated factors among preschool children in Gondar City, Northwest Ethiopia: a cross-sectional study. PLoS One 12, e0182511. 28787013 10.1371/journal.pone.0182511 PMC5546603

[46] Imamura F, Micha R, Khatibzadeh S, (2015) Dietary quality among men and women in 187 countries in 1990 and 2010: a systematic assessment. Lancet Global Health 3, e132–e142. 25701991 10.1016/S2214-109X(14)70381-X PMC4342410

[47] Jansen PW, Tharner A, Van Der Ende J, (2014) Feeding practices and child weight: is the association bidirectional in preschool children? Am J Clin Nutr 100, 1329–1336. 25332330 10.3945/ajcn.114.088922

[48] Afonso L, Lopes C, Severo M, (2016) Bidirectional association between parental child-feeding practices and body mass index at 4 and 7 y of age. Am J Clin Nutr 103, 861–867. 26843159 10.3945/ajcn.115.120824

[49] McKee C, Long L, Southward LH, (2016) The role of parental misperception of child's body weight in childhood obesity. J Pediatr Nurs 31, 196–203. 26521022 10.1016/j.pedn.2015.10.003

[50] Warkentin S, Mais LA, Maria do Rosário D, (2018) Factors associated with parental underestimation of child's weight status. J Pediatr 94, 162–169. 10.1016/j.jped.2017.05.010 28826796

[51] Spruijt-Metz D, Lindquist CH, Birch LL, (2002) Relation between mothers’ child-feeding practices and children's adiposity. Am J Clin Nutr 75, 581–586. 11864866 10.1093/ajcn/75.3.581

[52] de Lauzon-Guillain B, Musher-Eizenman D, Leporc E, (2009) Parental feeding practices in the United States and in France: relationships with child's characteristics and parent's eating behavior. J Am Diet Assoc 109, 1064–1069. 19465189 10.1016/j.jada.2009.03.008

[53] Faith MS, Berkowitz RI, Stallings VA, (2004) Parental feeding attitudes and styles and child body mass index: prospective analysis of a gene-environment interaction. Pediatrics 114, e429–e436. 15466068 10.1542/peds.2003-1075-L

[54] Marco PL, Valério ID, Zanatti C, (2020) Systematic review: symptoms of parental depression and anxiety and offspring overweight. Rev Saude Publica 54, 49. 32491095 10.11606/s1518-8787.2020054001731 PMC7234214

[55] Rodgers RF, Paxton SJ, McLean SA, (2014) Maternal negative affect is associated with emotional feeding practices and emotional eating in young children. Appetite 80, 242–247. 24859640 10.1016/j.appet.2014.05.022

[56] Goulding AN, Rosenblum KL, Miller AL, (2014) Associations between maternal depressive symptoms and child feeding practices in a cross-sectional study of low-income mothers and their young children. Int J Behav Nutr Phys Act 11, 75. 24935753 10.1186/1479-5868-11-75 PMC4072610

[57] Innella N, McNaughton D, Schoeny M, (2019) Child temperament, maternal feeding practices, and parenting styles and their influence on obesogenic behaviors in Hispanic preschool children. J Sch Nurs 35, 287–298. 29699450 10.1177/1059840518771485

[58] Horn MG, Galloway AT, Webb RM, (2011) The role of child temperament in parental child feeding practices and attitudes using a sibling design. Appetite 57, 510–516. 21740941 10.1016/j.appet.2011.06.015

[59] Hafstad GS, Abebe DS, Torgersen L, (2013) Picky eating in preschool children: the predictive role of the child's temperament and mother's negative affectivity. Eat Behav 14, 274–277. 23910765 10.1016/j.eatbeh.2013.04.001

[60] Vereecken CA, Keukelier E & Maes L (2004) Influence of mother's educational level on food parenting practices and food habits of young children. Appetite 43, 93–103. 15262022 10.1016/j.appet.2004.04.002

[61] Hupkens CL, Knibbe RA, Van Otterloo AH, (2000) Eat it or leave it: educational differences in how mothers handle children's food dislikes. Ecol Food Nutr 39, 247–270.

[62] St-Onge M-P, Keller KL & Heymsfield SB (2003) Changes in childhood food consumption patterns: a cause for concern in light of increasing body weights. Am J Clin Nutr 78, 1068–1073. 14668265 10.1093/ajcn/78.6.1068

[63] Pallant J (2001) Checking the reliability of a scale. SPSS Survival Manual. Version.

